# Effects of exogenous taurine supplementation on the growth, antioxidant capacity, intestine immunity, and resistance against *Streptococcus agalactiae* in juvenile golden pompano (*Trachinotus ovatus*) fed with a low-fishmeal diet

**DOI:** 10.3389/fimmu.2022.1036821

**Published:** 2022-10-14

**Authors:** Jia-Xing Liu, Hua-Yang Guo, Ke-Cheng Zhu, Bao-Suo Liu, Nan Zhang, Dian-Chang Zhang

**Affiliations:** ^1^ Key Laboratory of South China Sea Fishery Resources Exploitation and Utilization, Ministry of Agriculture and Rural Affairs, South China Sea Fisheries Research Institute, Chinese Academy of Fishery Sciences, Guangzhou, China; ^2^ College of Fisheries, Dalian Ocean University, Dalian, China; ^3^ Sanya Tropical Fisheries Research Institute, Sanya, China; ^4^ Guangdong Provincial Engineer Technology Research Center of Marine Biological Seed Industry, Guangzhou, China

**Keywords:** *Trachinotus ovatus*, taurine, intestine immunity, growth, antioxidant capacity, *Streptococcus agalactiae*

## Abstract

Taurine has various biological functions in fish, playing an essential role in growth, resistance to oxidative stress, and intestine immunity. Here, we evaluated the effects of exogenous taurine added to low-fishmeal diets on the growth, anti-oxidative stress, intestine immunity, and *Streptococcus agalactiae* resistance in juvenile golden pompano (*Trachinotus ovatus*). Our study showed that exogenous taurine supplementation of 1.2% (T3 group) greatly enhanced the weight gain rate and specific growth rate (SGR) of juvenile golden pompano, significantly upregulating growth-related factor expression in the brain and liver, as well as the levels of growth-related parameters in the serum. Polynomial regression analysis using SGR estimated the optimal dietary taurine level for golden pompano at 1.18%. Moderate exogenous taurine also increased the muscular thickness and villus length within the intestine, maintained intestinal physical barrier stability, activated the Nrf2/Keap-1/HO-1 signaling pathway, increased intestinal antioxidant enzyme gene expression and antioxidant enzyme activity in the serum, and upregulated immunoglobulin and complement levels in parallel with declining reactive oxygen species (ROS) levels in the serum. Antioxidant factor expression was also upregulated in the intestine. Furthermore, supplementation suppressed NF-κB signaling and intestinal pro-inflammatory cytokine gene expression, increased anti-inflammatory cytokine gene expression, and improved intestine immunity. Finally, taurine supplementation improved the survival rate of golden pompano challenged with *S. agalactiae*. Overall, our findings provide additional information and support for the rational use of taurine in healthy aquatic animal farming.

## Introduction

As a source of high-quality protein for aquaculture, the limited availability of fishmeal has led to soaring prices, resulting in its partial replacement with plant and animal protein for large-scale economic fish farming (1–5). Currently, fishmeal substitutes include animal (including chicken (1) and feather meal (2)) and plant (including soybean meal (3), fermented soybean meal (4), soybean protein concentrate (5), and corn gluten meal (2)) protein. However, replacing fishmeal with other plant and animal proteins in excess can cause a range of negative effects on aquatic animals. For example, low-fishmeal ratios can cause reduced growth performance (6), altered intestinal microflora (7), structural damage to the intestine (8), oxidative stress (9), and reduced intestine immunity (10, 11) in fish, leading to greater susceptibility to bacteria and viruses. Therefore, it is essential to explore ways to mitigate the negative effects of low-fishmeal feeds on growth performance, antioxidant capacity, and intestine immunity of aquatic animals.

Taurine, chemically known as 2-aminoethanesulfonic acid (C_2_H_7_NO_3_S), is a β-sulfur-containing amino acid that promotes growth, improves antioxidant capacity, and protects against inflammation. As a functional amino acid, taurine has been widely studied and applied in aquatic animal nutrition (12). Numerous studies have shown that exogenous taurine in low-fishmeal diets can effectively enhance the growth of *Monopteros albus* (13, 14), *Pagrus major* (15), *Dicentrarchus labrax* (16), and *Rachycentron canadum* (17). Taurine is highly effective against oxidative stress, reducing the oxidative stress effect of Cd on the liver and kidneys of *Clarias batrachus* (18). Studies on *Ictalurus punctatus* (19) showed that taurine could significantly upregulate the mRNA expression of antioxidant enzymes through the Nrf2/Keap-1 signaling pathway to attenuate intestinal oxidative damage and effectively avoid weakened intestine immunity caused by oxidative damage.

Intestine immunity plays a vital role in the regulation of immune homeostasis. Therefore, intestinal health is essential for healthy aquatic animal husbandry (20). The intestinal health of aquatic animals depends on the combined effects of the intestinal physical barrier, antioxidant capacity, and immune barrier (21). Taurine was shown to improve intestinal barrier integrity, increasing villus length and muscular thickness in *M. albus* (13) under a high-fat diet. This resulted in a more regular arrangement of the intestinal villi, which was beneficial for maintaining intestinal health. Taurine is also a good facilitator of intestine immunity in aquatic animals. Exogenous taurine was effective in reducing the intestinal inflammatory response and improving intestinal health in *I. punctatus* (19), *Ctenopharyngodon idella* (22), and *D. labrax* (23).

Golden pompano (*Trachinotus ovatus*) has a wide distribution, high adaptability, and tasty meat. Owing to the maturation of artificial broodstock and culture techniques, it has become one of the most essential marine fish in China for large-scale culture (24, 25). However, golden pompano fed low-fishmeal feed are prone to decreased growth performance, oxidative stress, intestinal structural damage, dysbiosis, and reduced intestine immunity (26–28). In recent years, frequent outbreaks of bacterial diseases during culture have occurred, most often caused by *Streptococcus agalactiae* (29, 30), *Vibrio harveyi* (31–33), and *Vibrio vulnificus* (34, 35) infections, resulting in significant economic losses. Therefore, in the context of a shortage of fishmeal resources and rising prices, the development of feed additives that promote the growth of golden pompano, maintain intestinal health, and improve intestine immunity are vital for its healthy culture.

This study aimed to determine the effects of exogenous taurine added to low-fishmeal feeds on growth, intestinal health, and resistance against *S. agalactiae* in juvenile golden pompano to provide a theoretical basis for taurine supplementation during their culture.

## Materials and methods

### Ethical statement

All experiments in this study were approved by the Animal Care and Use Committee of the South China Sea Fisheries Research Institute, Chinese Academy of Fishery Sciences (No. SCSFRI96-253) and performed according to the regulations and guidelines established by this committee.

### Experimental diets

Taurine with a purity of 99.99% was purchased from Guangzhou Nutriera Biotechnology Co., Ltd. (Guangzhou, China). The feed formulations used in these experiments are listed in **Table 1**. Five isonitrogenous and isoenergetic diets were formulated according to the nutritional requirements of the golden pompano. Protein sources included fishmeal, chicken meal, soybean protein concentrate, fermented soybean meal, and corn protein meal. Lipid sources included fish and soybean oils. Taurine was added at 0.00% (T0), 0.40% (T1), 0.80% (T2), 1.20% (T3), and 1.60% (T4), respectively. First, all ingredients were added to the powder through a 40-mesh sieve. Second, fish oil, soybean oil, and water were slowly added and mixed to create a sinkable pellet feed using the Valva-60D-III twin-screw puffing and granulating machine (Valva Machinery Co., Ltd., Guangzhou, China). Finally, the diets were dried in an oven at 45°C until the moisture content was less than 10% and saved at 4°C (27). The amino acid composition of experimental diets is shown in **Table 2**.

**Table 1:** Formulation and nutrition level of the experimental diets (% dry matter basis).

Parameters	Group				
	T0	T1	T2	T3	T4
Ingredients (%)					
Fishmeal ^a^	20.00	20.00	20.00	20.00	20.00
Chicken meal ^a^	10.00	10.00	10.00	10.00	10.00
Soy protein concentrate ^a^	10.00	10.00	10.00	10.00	10.00
Squid paste	5.00	5.00	5.00	5.00	5.00
Soybean meal ^a^	12.00	12.00	12.00	12.00	12.00
Fermented soybean meal ^a^	5.00	5.00	5.00	5.00	5.00
Corn gluten meal ^a^	6.00	6.00	6.00	6.00	6.00
High gluten flour ^a^	18.37	17.97	17.57	17.17	16.77
Fish oil ^a^	6.00	6.00	6.00	6.00	6.00
Soybean oil ^a^	3.00	3.00	3.00	3.00	3.00
Ca(H_2_PO_4_)_2_ ^a^	1.50	1.50	1.50	1.50	1.50
Choline chloride ^a^	0.30	0.30	0.30	0.30	0.30
Vitamin mix ^a,b^	1.00	1.00	1.00	1.00	1.00
Mineral mix ^a,c^	1.00	1.00	1.00	1.00	1.00
L-lysine monohydrochloride ^a^	0.50	0.50	0.50	0.50	0.50
DL-Methionine ^a^	0.20	0.20	0.20	0.20	0.20
Threonine ^a^	0.10	0.10	0.10	0.10	0.10
Ethoxyquin ^a^	0.03	0.03	0.03	0.03	0.03
Taurine ^a^	0.00	0.40	0.80	1.20	1.60
Nutrition level ^d^					
Crude Protein (% dry matter)	42.79	42.74	42.69	42.63	42.58
Crude Lipid (% dry matter)	13.42	13.40	13.38	13.37	13.35
Moisture (% dry matter)	10.15	10.76	11.24	10.98	11.32
Ash (% dry matter)	8.53	8.65	8.33	8.71	8.39
Taurine	0.27	0.68	1.09	1.49	1.87

a Ingredients are provided by Guangzhou Nutriera Biotechnology Co., Ltd.

b Vitamin mix provides the following (Per kilogram content): vitamin A (8×10^6^ IU), vitamin D3 (2×10^6^ IU), vitamin E 40 000 mg, vitamin B 17 000 mg, vitamin B6 12 000 mg, vitamin B12 100 mg, vitamin K3 10 000 mg, D-pantothenic acid 35 000 mg, folic acid 1 000 mg, nicotinamide 90 000 mg, Biotin 200 mg, inositol 80 000 mg.

c Mineral provides the following (Per kilogram content): Fe 10 000 mg, Cu 1 200 mg, Zn 7 000 mg, Mn 5 500 mg, Co 250 mg, I2 250 mg, Se 50 mg, K 60 000 mg, Na 24 000 mg, Mg 60 000 mg

d Nutrition level is measured.

**Table 2:** Amino acid composition of the experimental diets(g·100g^−1^).

Parameters	Group				
	T0	T1	T2	T3	T4
Aspartic acid	4.21	4.94	4.94	4.75	4.18
Threonine	1.93	2.26	1.94	2.16	1.90
Serine	2.08	2.49	2.46	2.30	2.05
Glutamic acid	8.38	10.2	8.94	9.64	8.54
Glycine	3.21	3.57	3.63	3.58	3.16
Alanine	2.86	3.30	2.82	3.21	2.81
Cysteine	0.56	0.50	0.56	0.56	0.46
Valine	1.99	2.27	2.13	2.34	2.03
Methionine	1.10	1.28	0.20	1.14	0.98
Isoleucine	1.65	1.90	1.77	1.96	1.74
Leucine	3.94	4.61	3.92	4.44	3.91
Tyrosine	1.25	1.45	1.30	1.43	1.30
Phenylalanine	2.14	2.54	2.47	2.44	2.16
Lysine	3.18	3.67	3.51	3.59	3.15
Histidine	1.06	1.24	1.20	1.23	1.05
Arginine	2.71	3.18	2.60	3.10	2.73
Proline	3.14	3.57	3.57	3.37	3.04
Taurine	0.27	0.68	1.09	1.49	1.87

### Experimental procedure

The culture experiments were conducted in offshore cages (100 cm × 100 cm × 200 cm), in the Dapeng New District of Shenzhen, China. Juvenile golden pompano and *S. agalactiae* were obtained from our laboratory. All test fish were reared on T0 group feed without exogenous taurine for seven days before the start of the experiment. Subsequently, 1,050 fish (10.05 ± 0.05 g) were divided into 5 diet groups and placed in 15 cages (3 cages per diet group, 70 fish per cage). Each diet was administered four times daily, at 8:00, 10:00, 14:00, and 16:00 (feed ratio of 2:2:3:3), until apparent satiation for a total of eight weeks. Feeding status of the golden pompano was observed daily, and water quality conditions, water temperature, dissolved oxygen concentration, pH, and salinity were measured daily. Although several fish died during the trial, there were no major disease outbreaks.

### Sample collection

The number of golden pompano in each cage was counted at the end of the culture period. After 24 h of fasting, nine fish per cage were randomly taken, placed in buckets containing eugenol (100–200 mg/L; Shanghai Medical Instruments Co., Ltd., Shanghai, China) for anesthesia, and then weighed. First, three fish per cage were snap-frozen in liquid nitrogen and then transferred to -80°C to analyze the routine nutrient composition. Second, blood was drawn from the three fish per cage using 2 mL syringes treated with 1% sodium heparin solution and centrifuged (3,500 × *g*, 4°C) for 10 min, after which the supernatant was transferred into a 1.5 mL cryotube and stored at -80°C for analysis of serum, growth, antioxidant, and immunological parameters. After collecting the serum, the brain, liver, and intestine of golden pompano were quickly collected, rapidly frozen in liquid nitrogen, transferred to -80°C, and stored for use in RNA extraction and gene expression analysis. Finally, the midguts of the remaining three fish per cage were removed and placed in a sampling bottle containing 4% paraformaldehyde solution and transferred to 4°C after 24 h for storage and used for histological analysis.

### Growth performance

The parameters of growth performance were calculated as per the following formulas:


Weight gain rate (WGR,%)=100 x (final fish body weight-initial fish body weight)/initial fish body weight

Specific growth rate (SGR,%/ day)=100 x (ln final fish body weight-ln initial fish body weight)/number of days

Feed conversion ratio (FCR)=dry diet intake/net weight gain

$${\text{Condition factor (CF,g/cm}}^{3})=100\;{\text{x final fish body weight/final fish body length}}^{3}$$

Survival rate (SR,%)=100 x final fish number/30

Hepatosomatic index (HSI,%)=100 x liver weight/final fish body weight

Viscerasomatic index (VSI,%)=100 x viscera weight/final fish body weight

Feed intake (FI,%/day)=100 x dry diet intake/[initial fish body weight + final fishbody weight)/2]/number of days

### Organism composition

The organism composition of whole fish analysis was performed as follows. The content of crude protein and crude lipid were determined using Kjeldahl nitrogen determination and Soxhlet extraction, respectively. The moisture and ash content of whole fish was detected *via* constant weight and muffle furnace cautery method, respectively (AOAC, 2000).

### Serum biochemical and immunological parameters analysis

The serum levels of growth hormone (GH), triiodothyronine, thyroxine, insulin-like growth factor receptor-1 (IGF-1), and insulin-like growth factor receptor-2 (IGF-2) were determined using kits (Jian Cheng Bioengineering Institute, Nanjing, China). The total antioxidant capacity (T-AOC), catalase (CAT), glutathione peroxidase (GSH-PX), superoxide dismutase (SOD), and lysozyme (LZM) activities, as well as the levels of malondialdehyde (MDA), reactive oxygen species (ROS), complement 3 (C3), complement 4 (C4), and immunoglobulins (IgA, IgG, and IgM) assays were performed with the respective kits (Beijing Sin-Uk Institute of Biological Technology, Beijing, China).

### Midgut histological observation

As previously described by Ding et al. (36), we subjected golden pompano midguts to hematoxylin and eosin (H&E) staining to observe histological structures. First, the midgut was fixed in 4% paraformaldehyde solution for 24 h and dehydrated *via* ethanol grading. Samples were then washed in xylene and paraffin-embedded. After the paraffin wax was completely solidified, it was cut into 5-μm-thick sections using a slicer. The sections were transferred to a water bath at 40°C, and the paraffin wax was melted, allowing the samples to adhere to the slides, which were then dried in an oven at 40°C. They were removed after 24 h, H&E staining and sealing were used to prepare intestinal histological sections. Histological sections were observed under a 200 × light microscope (Leica, Wetzlar, Germany) and intestinal morphological parameters were measured by Image-Pro Plus 6.0 software (National Institutes of Health, Bethesda, USA). The field of view of each slide was divided equally into eight sections, and the length of intact intestinal villi, muscular thickness, and goblet cell number on each intestinal villus were randomly measured in each section. The mean values were used for analysis and preparing graphs in GraphPad Prism 8 (San Diego, California, USA).

### Quantitative reverse-transcription PCR

To evaluate the regulatory effects of taurine on growth, intestine immunity, and resistance to oxidative stress in the golden pompano, we selected the following genes for qRT-PCR. First, we analyzed *growth hormone* (*GH*) and *neuropeptide Y* (*NPY*) mRNA expression in the brain. We then determined *insulin-like growth factor receptor-1* (*IGF-1*) and *insulin-like growth factor receptor-2* (*IGF-2*) mRNA expression in the liver. Finally, *CAT*, *GSH-PX*, *SOD*, *nf-e2-related nuclear factor2* (*Nrf2*), *Kelch-like ECH-associated protein-1* (*Keap-1*), *heme oxygenase-1* (*HO-1*), *nuclear factor kappa B* (*NF-κB*), *inhibitor protein-κB* (*IκB*), *IκB kinase* (*IKK*), *tumor necrosis factor-α* (*TNF-α*), *interleukin 1β* (*IL-1β*), *interleukin 8* (*IL-8*), and *interleukin 10* (*IL-10*) mRNA expression were determined in the intestine. *EF-1α* was selected as the housekeeping gene (37), and the primer sources for all tested genes are shown in **Table 3**, where the primers of the *NPY* were designed by the Primer Premier 6 (Premier Biosoft, Canada).

**Table 3:** Real-time PCR primer sequences.

Primers	Forward primer sequences (5′-3′)	Reverse primer sequences (5′-3′)	Source
*GH*	CAGCCAATCACAGACAGCC	GGAACTCCCAAGACTCCACTAA	Liu et al. (38)
*IGF-1*	CGCTAAATCTCACTTCTCCAAAA	CTCATCAAACCCTTAAACACCAC	Yang et al. (39)
*IGF-2*	CAATCTCTCCAACCAAATAACCC	CTTTTTTTCTCCCTCCAAACTCT	Yang et al. (39)
*NPY*	AAAAGACCACCCTGCCTCTG	ATGGCTAAGGAGGAGGGGTT	GenBank: OP292223
*CAT*	GGATGGACAGCCTTCAAGTTCTCG	TGGACCGTTACAACAGTGCAGATG	Liu et al. (24)
*SOD*	CCTCATCCCCCTGCTTGGTA	CCAGGGAGGGATGAGAGGTG	Liu et al. (24)
*GSH-PX*	GCTGAGAGGCTGGTGCAAGTG	TTCAAGCGTTACAGCAGGAGGTTC	Liu et al. (24)
*HO-1*	AGAAGATTCAGACAGCAGCAGAACAG	TCATACAGCGAGCACAGGAGGAG	Xie et al. (40)
*Nrf2*	TTGCCTGGACACAACTGCTGTTAC	TCTGTGACGGTGGCAGTGGAC	Liu et al. (41)
*Keap-1*	CAGATAGACAGCGTGGTGAAGGC	GACAGTGAGACAGGTTGAAGAACTCC	Liu et al. (41)
*IL-1β*	CGGACTCGAACGTGGTCACATTC	AATATGGAAGGCAACCGTGCTCAG	Liu et al. (24)
*IL-8*	CCGATCAACAGGGACTTCAA	GAGGACCGAGGGTTCAGACAG	Zhang et al. (42)
*IL-10*	AGTCAGTCTCCACCCCCATCTT	GCCCACTGGAGTTCAGATGCT	Zhang et al. (42)
*TNF-α*	GCTCCTCACCCACACCATCA	CCAAAGTAGACCTGCCCAGACT	Liu et al. (24)
*NF-κB*	CGTGAGGTCAGCGAGCCAATG	ATGTGCCGTCTATCTTGTGGAATGG	Liu et al. (24)
*IKK*	CCTGGAGAACTGCTGTGGAATGAG	ATGGAGGTAGGTCAGAGCCGAAG	Liu et al. (24)
*IκB*	GCTGGTCCATTGCCTCCTGAAC	GTGCCGTCTTCTCGTACAACTGG	Liu et al. (24)
*EF-1α*	AAGCCAGGTATGGTTGTCAACTTT	CGTGGTGCATCTCCACAGACT	Ma et al. (37)

In this experiment, RNA was extracted from the brain, liver, and intestine by the HiPure Universal RNA Mini kit (Magen Biotech Co., Ltd., Guangzhou, China). RNA mass and concentration were determined using NanoDrop 2000 (Thermo Scientific, USA) and 1% agarose gel electrophoresis. cDNA was prepared using a PrimeScript™ RT kit and gDNA Eraser (TaKaRa, Dalian, China). qRT-PCR was performed using the SYBR^®^ Green Premix Pro Taq HS qPCR Kit. The reaction conditions for qPCR were as described by Ma et al. (37). To eliminate the effect of chance, each sample was analyzed four times in duplicate, and three results were selected to calculate the relative expression levels of the target gene *via* the 2^-∆∆CT^ method (43).

### Streptococcus agalactiae challenge

According to a previous study by Gao et al. (25), the LC50 of golden pompano after 120 h of *S. agalactiae* challenge corresponded to a *S. agalactiae* concentration of 2.0 × 10^7^ CFU/fish. After eight weeks of feeding, 20 similarly sized and healthy fish per cage were randomly taken, and 200 μL of bacterial suspension at the concentration of 2.0 × 10^7^ CFU/fish was intraperitoneally injected into each golden pompano using a sterile syringe. The same water temperature, pH, salinity, and dissolved oxygen mass concentration used during the culture trial were maintained throughout the 120 h challenge. The fish in each group were fed under the same conditions as the culture trial, with satiety feeding four times a day (8:00, 10:00, 14:00, and 16:00), and the mortality of each group was recorded every 12 h. The survival rate (%) was calculated at the end of the challenge.

### Statistical analysis

One-way analysis of variance (ANOVA) was performed on the experimental data using SPSS 26.0. Tukey’s test was used for multiple comparisons when differences were significant (*P* < 0.05). The results are expressed as mean ± standard deviation (mean ± SD). The Kaplan–Meier method was used to construct survival curves after *S. agalactiae* challenge, and differences in feed between the control and test groups were compared using the log-rank test.

## Results

### Growth performance

**Figure 1:**
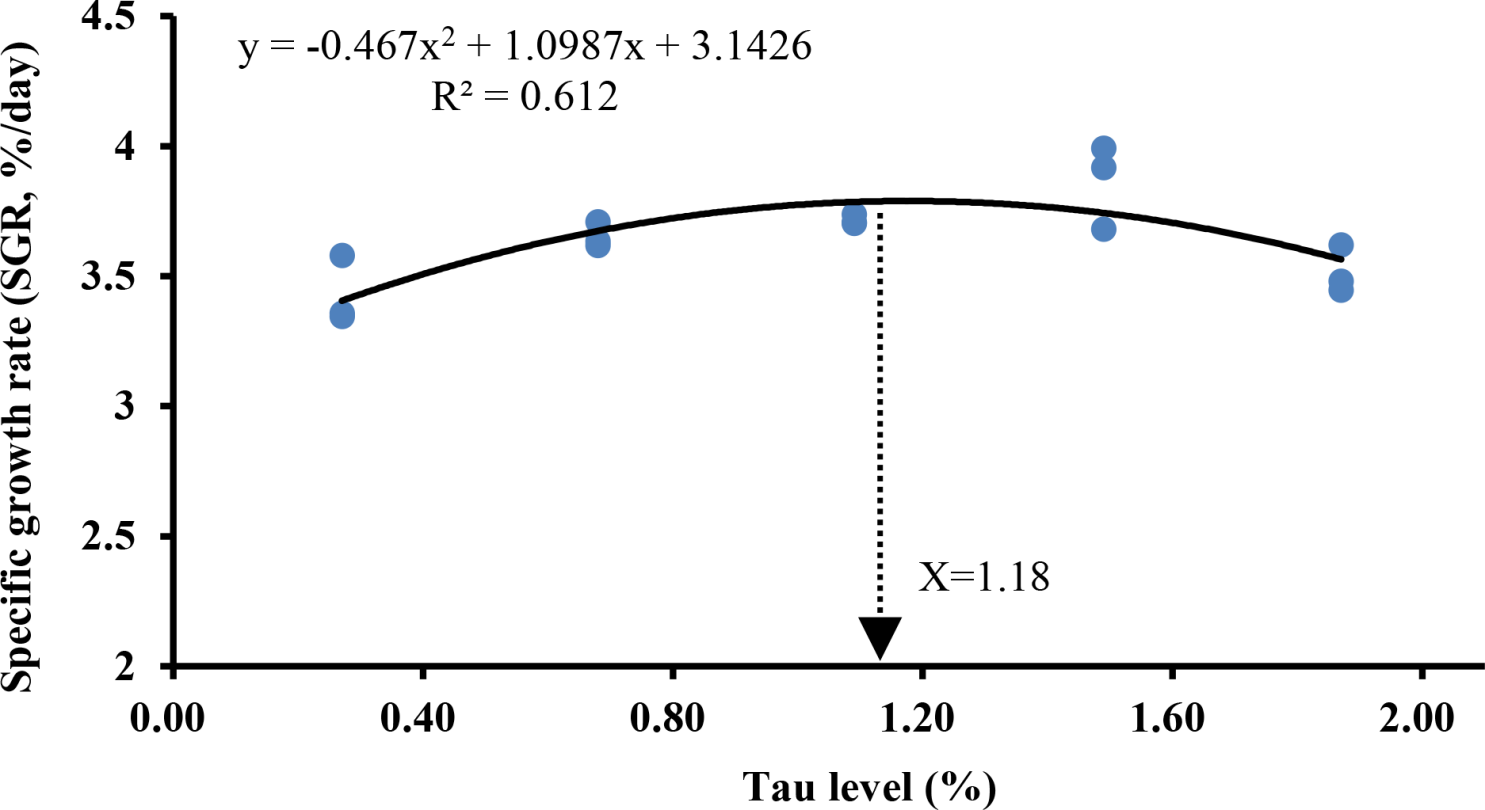
Estimation of the optimal dietary taurine level for *T. ovatus* by means of polynomial regression analysis using the SGR.

As can be seen in **Table 4**, with the increase of exogenous taurine supplementation, the FBW, WGR, and SGR were greatly increased (*P* < 0.05). Especially in the T3 group, all indexes contained the highest values, indicating that exogenous taurine might greatly enhance the growth performance of golden pompano. In addition, FI, FCR, and HSI also demonstrated a significant decline with increasing exogenous taurine. However, exogenous taurine supplementation had no effect on SR, VSI, and CF (*P* > 0.05). As can be seen in **Figure 1**, the optimal taurine level in the diet of golden pompano was estimated at 1.18% *via* polynomial regression analysis using SGR.

**Table 4:** Growth performance of *T. ovatus* fed diets with different dose taurine supplementation after 8 weeks.

Parameters	Group					*P* valve
	T0	T1	T2	T3	T4	
SR (%)	95.24 ± 0.82	95.24 ± 0.82	96.67 ± 0.82	96.67 ± 2.18	95.71 ± 1.43	0.507
IBW (g)	10.15 ± 0.02	10.12 ± 0.05	10.11 ± 0.05	10.05 ± 0.03	10.08 ± 0.04	0.784
FBW (g)	68.64 ± 5.22^a^	77.91 ± 2.09^bc^	80.79 ± 0.97^cd^	87.98 ± 7.83^d^	72.10 ± 3.85^ab^	<0.001
WGR (%)	583.03 ± 51.95^a^	675.23 ± 20.82^ab^	703.91 ± 9.64^ab^	775.40 ± 77.92^b^	617.41 ± 38.30^a^	<0.001
SGR (%/day)	3.43 ± 0.13^a^	3.65 ± 0.05^bc^	3.71 ± 0.02^bc^	3.86 ± 0.16^c^	3.51 ± 0.09^ab^	<0.001
FI (%/day)	1.42 ± 0.05^b^	1.20 ± 0.02^a^	1.30 ± 0.01^ab^	1.24 ± 0.06^ab^	1.32 ± 0.03^ab^	<0.001
FCR (%)	2.15 ± 0.18^b^	1.75 ± 0.05^a^	1.87 ± 0.02^ab^	1.76 ± 0.19^a^	1.96 ± 0.11^ab^	<0.001
HSI (%)	1.28 ± 0.07^b^	1.07 ± 0.08^a^	1.05 ± 0.08^a^	1.00 ± 0.09^a^	1.03 ± 0.07^a^	<0.001
VSI (%)	5.50 ± 0.34	5.37 ± 0.40	5.18 ± 0.52	5.55 ± 0.16	5.69 ± 0.65	0.170
CF (g/cm^3^)	2.69 ± 0.31	3.36 ± 0.56	3.27 ± 0.66	2.81 ± 0.47	2.83 ± 0.52	0.062

values in the same row with different superscripts are significantly different (P < 0.05).

### Organism composition

**Table 5** showed that exogenous taurine greatly increased crude protein content in the T3 group compared to that in the control group (*P* < 0.05). The highest moisture content was observed in the control group, which was extremely higher compared to all other groups (*P* < 0.05). The crude lipid and ash contents were not influenced by exogenous taurine (*P* > 0.05).

**Table 5:** Effect of dietary taurine level on organism composition of *T. ovatus.*

Parameters	Group					*P* valve
	T0	T1	T2	T3	T4	
Crude protein (g·100g^-1^)	17.37 ± 0.15^a^	17.40 ± 0.10^a^	18.00 ± 0.26^ab^	18.17 ± 0.45^b^	17.30 ± 0.26^a^	0.008
Crude lipid (g·100g^-1^)	6.03 ± 0.31	5.80 ± 0.20	5.97 ± 0.38	5.97 ± 0.31	5.93 ± 0.31	0.909
Moisture (g·100g^-1^)	73.30 ± 0.59^b^	70.67 ± 0.40^a^	70.83 ± 0.70^a^	69.87 ± 0.45^a^	69.80 ± 0.10^a^	<0.001
Ash (g·100g^-1^)	4.30 ± 0.10	4.09 ± 0.17	4.06 ± 0.23	4.17 ± 0.17	4.07 ± 0.15	0.435

values in the same row with different superscripts are significantly different (P < 0.05).

### Serum growth-related parameters

**Figure 2:**
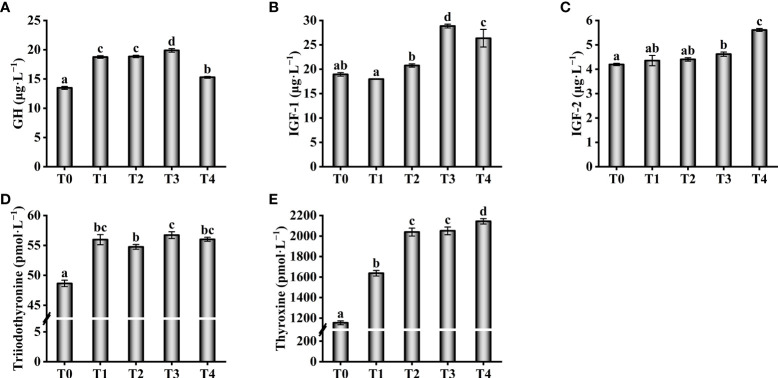
Effect of growth-related parameters such as GH **(A)**, IGF-1 **(B)**, IGF-2 **(C)**, Triiodothyronine **(D)** and Thyroxine **(E)** in the serum of *T. ovatus* fed diets with different dose taurine supplementation after 8 weeks. Mean values (n = 9) within values in the picture above with different superscripts are significantly different (*P* < 0.05).

With an increase in taurine content, the GH levels in serum showed a trend of increase (0.4–1.2%) and subsequent decrease (1.2–1.6%, **Figure 2A**). The lowest serum GH level was identified in the control group, which was much lower than those of the experimental groups (*P* < 0.05). The levels of IGF-1 and IGF-2 in serum increased with increasing exogenous taurine supplementation and were significantly higher in the T3 and T4 than in the other groups (*P* < 0.05, **Figures 2B, C**). The lowest triiodothyronine and thyroxine levels were observed in the control group, which were significantly lower than those in the other groups (*P* < 0.05, **Figures 2D, E**).

### Growth-related genes

**Figure 3:**
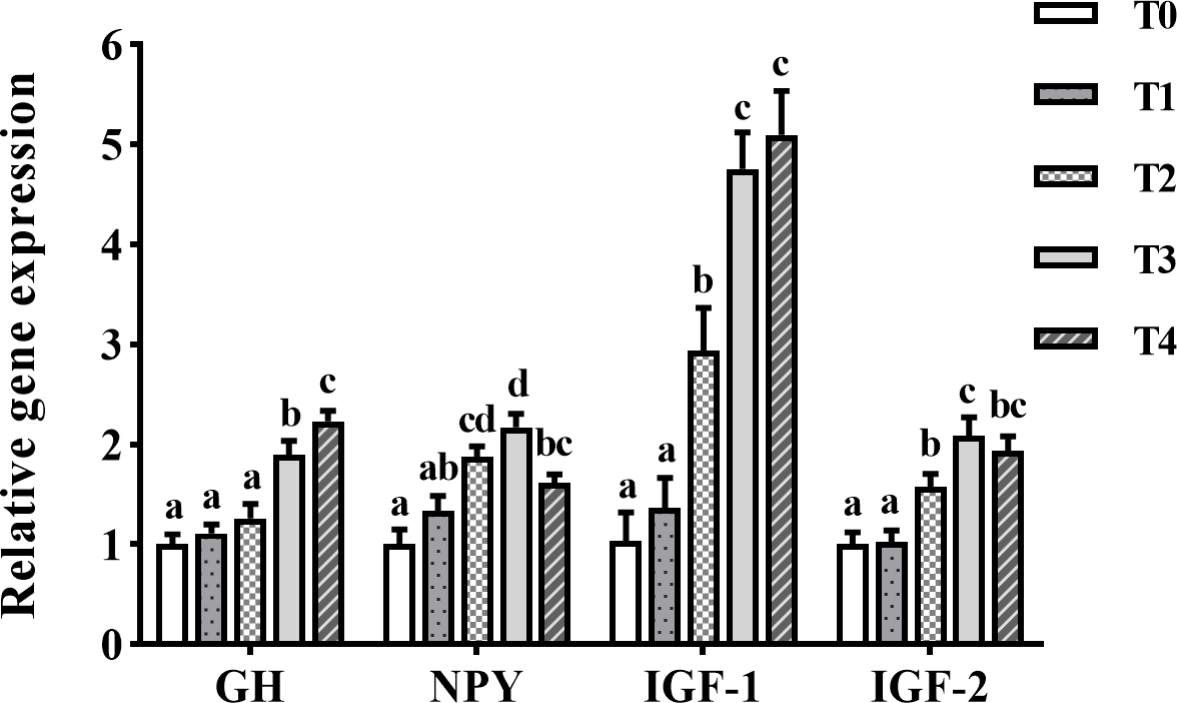
The expression profiles of growth-related genes in the liver and the brain of *T. ovatus* fed diets with different dose taurine supplementation after 8 weeks. Mean values (n = 9) within values in the picture above with different superscripts are significantly different (*P* < 0.05).

*GH* mRNA levels in the brain of golden pompano greatly increased with the increase of exogenous taurine supplementation (**Figure 3**). *GH* mRNA expression was greatly higher in the T3 and T4 groups than in the control group (*P* < 0.05). The *NPY* mRNA levels in the brain of golden pompano showed a trend of increasing (0–1.2%) and then decreasing (1.2–1.6%) with the increase of exogenous taurine supplementation. T3 fish exhibited the highest *NPY* expression, which was remarkably higher than the expression of the control group (*P* < 0.05). *IGF-1* and *IGF-2* expression in the liver showed a trend of increasing with the addition of exogenous taurine supplementation. The expression of *IGF-1* and *IGF-2* in T3 and T4 fish was the highest and much higher compared with theT0 fish (*P* < 0.05).

### Serum antioxidant capacity and non-specific immune parameters

**Figure 4:**
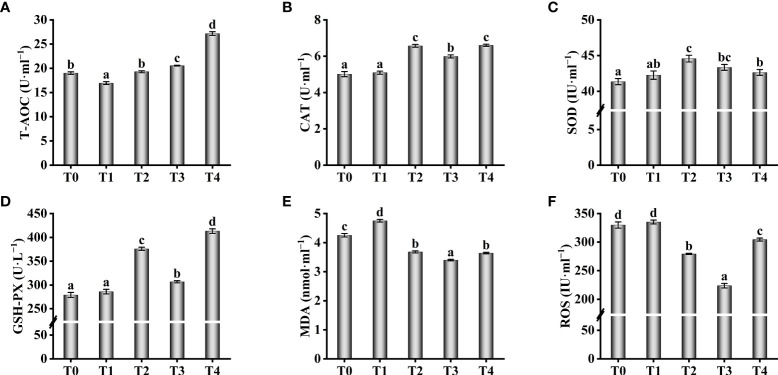
Effect of antioxidant capability such as T-AOC **(A)**, CAT **(B)**, SOD **(C)**, GSH-PX **(D)**, MDA **(E)** and ROS **(F)** in the serum of *T. ovatus* fed diets with different dose taurine supplementation after 8 weeks. Mean values (n = 9) within values in the picture above with different superscripts are significantly different (*P* < 0.05).

**Figure 5:**
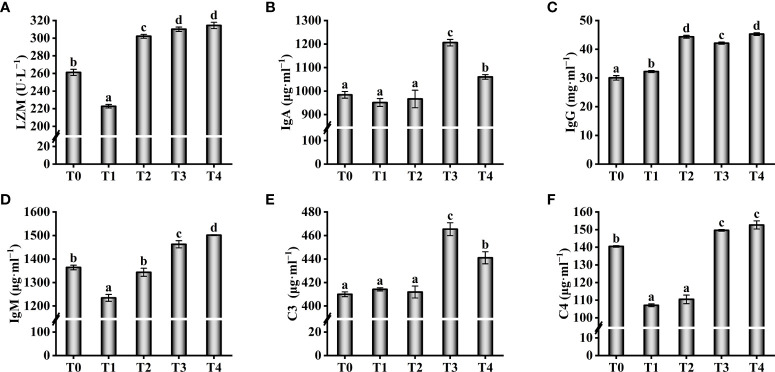
Effect of immunological parameters such as LZM **(A)**, IgA **(B)**, IgG **(C)**, IgM **(D)**, C3 **(E)**, C4 **(F)** in the serum of *T. ovatus* fed diets with different dose taurine supplementation after 8 weeks. Mean values (n = 9) within values in the picture above with different superscripts are significantly different (*P* < 0.05).

Antioxidant enzyme activity in serum increased with increasing exogenous taurine supplementation (**Figure 4**). The T-AOC enzyme activity in the T3 and T4 groups were greatly higher compared with theT0 group (*P* < 0.05, **Figure 4A**). The T-AOC enzyme activity in the T2 fish was similar to the control group, with no significant differences (*P* > 0.05). Still, the highest enzyme activities of CAT and SOD were found in the T2 fish, which were significantly higher compared with theT0 group (*P* < 0.05, **Figures 4B, C**). Compared with theT0 group, GSH-PX activity were higher while exogenous taurine supplementation from 0.8% to 1.6% (*P* < 0.05, **Figure 4D**), yet the enzymatic activity at 1.2% was markedly lower compared with 0.8% and 1.6% (*P* < 0.05).On the contrary, the MDA and ROS levels in the serum decreased with increasing exogenous taurine supplementation (**Figures 4E, F**). The MDA and ROS levels of the T3 group recorded the lowest value and were significantly lower than those of the control group (*P* < 0.05). **Figure 5** showed that the highest activity of LZM (**Figure 5A**) and the highest content of C4 (**Figure 5F**) were identified in T3 and T4 fish, which considerably higher than that in the control group (*P* < 0.05). IgA and C3 levels were significantly higher in the T3 group than in the other groups (*P* < 0.05, **Figures 5B, E**). IgM levels were higher in the T3 and T4 fish compared to those in the control group (*P* < 0.05, **Figure 5D**). IgG levels were higher at 0.8–1.6% exogenous taurine compared to the T0 group (*P* < 0.05), yet significantly lower at 1.2% compared to 0.8% and 1.6% exogenous taurine (*P* < 0.05, **Figure 5C**).

### Midgut histological observation

**Figure 6:**
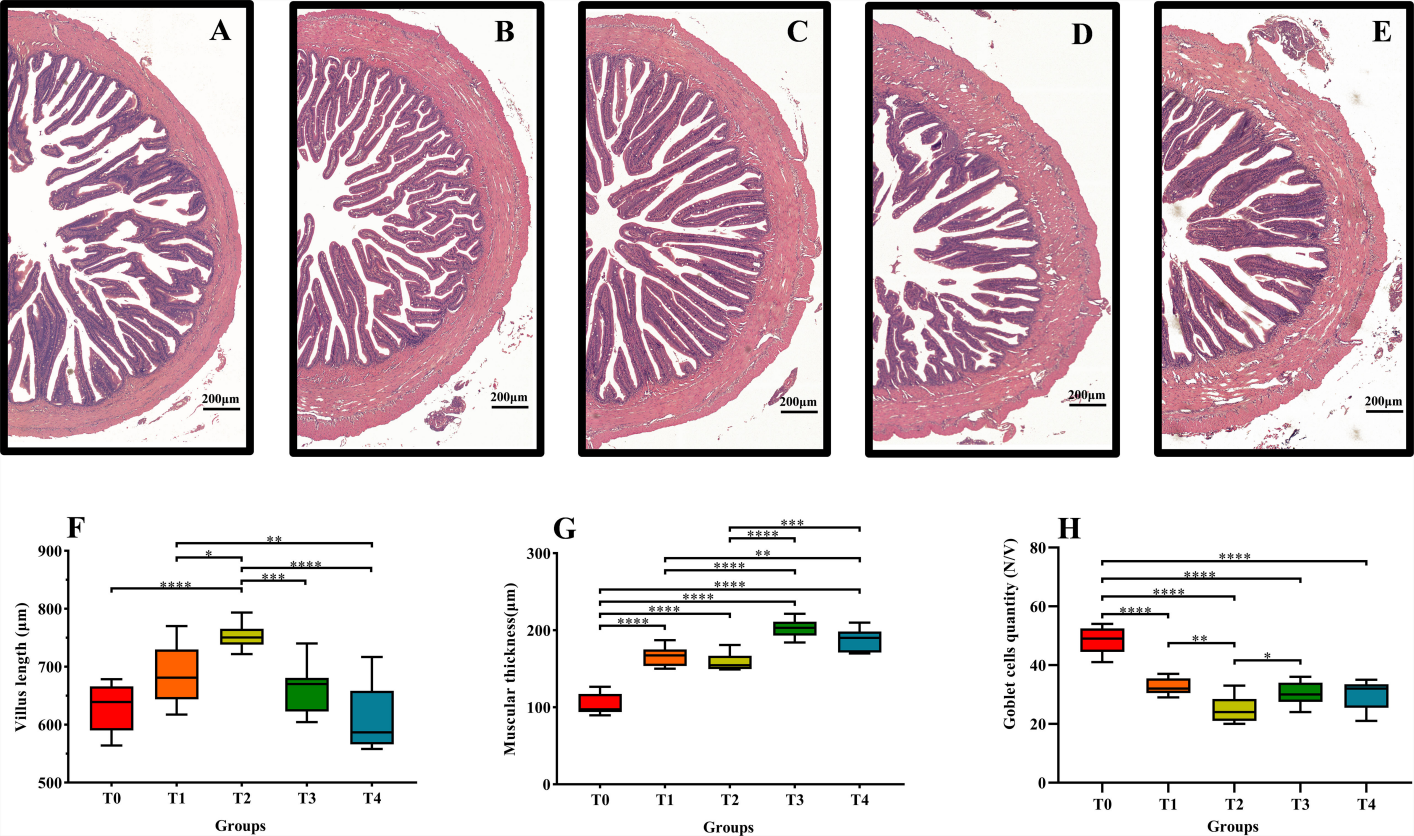
Effects of dietary taurine on mid-gut morphology of *T. ovatus*. **(A)**: 0% taurine; **(B)**: 0.40% taurine; **(C)**: 0.80% taurine; **(D)**: 1.20% taurine; **(E)**: 1.60% taurine. Scale bar: 200 μm. The villus length **(F)**, muscular thickness **(G)**, and goblet cells quantity **(H)** of mid-gut in *T. ovatus.* data are presented as mean ± SD (n = 9).Asterisks *, **, ***,and **** indicate statistically significant difference between treated group and control group at *P* < 0.05, *P* < 0.01 P < 0.001, and *P* < 0.0001, respectively.

**Figures 6A–E** are midgut sections of fish C0-C4, respectively. The villus length increased (0–0.8%) and then decreased (0.8–1.6%) with increasing exogenous taurine supplementation (**Figure 6F**). Villus length in the T2 group was the highest, and a significant increase occurred in the T2 group compared to that in the control group (*P* < 0.05). Muscle thickness increased with increasing taurine content (**Figure 6G**). The highest muscle thickness was observed in the T3 and T4 groups. The lowest muscle thickness recorded in the control group was lower than that in all other dietary groups, and the difference was highly significant (*P* < 0.05). By contrast, the number of goblet cells per intestinal villi exhibited a significant decline with increasing exogenous taurine supplementation; the control group recorded the highest number of goblet cells, and the difference was highly significant (*P* < 0.05, **Figure 6H**).

### Antioxidant enzyme expression in the intestine

**Figure 7:**
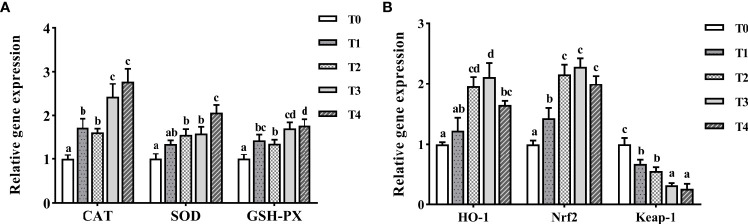
The expression profiles of antioxidant genes **(A)** and signaling pathway **(B)** in the intestine of *T. ovatus* fed diets with different dose taurine supplementation after 8 weeks. Mean values (n = 9) within values in the picture above with different superscripts are significantly different (*P* < 0.05).

As shown in **Figure 7A**, *CAT*, *SOD*, and *GSH-PX* mRNA levels increased with taurine content. The maximum expression of *CAT* and *GSH-PX* was observed with exogenous addition of 1.2–1.6% taurine. *SOD* mRNA level in the T4 group (1.6% taurine) was the highest, and much higher than that in the control group (*P* < 0.05). With the increasing taurine content, *HO-1* and *Nrf2* expression initially increased (0–1.2%) and then decreased (1.2–1.6%, **Figure 7B**). The maximum expression of *HO-1* and *Nrf2* was observed with the exogenous addition of 0.8–1.2% taurine and was significantly higher than that in the control fish (*P* < 0.05). Conversely, the higher the exogenous taurine addition, the lower the expression of *Keap-1* mRNA. The T0 group showed the highest *Keap-1* expression and was greatly higher than other groups (*P* < 0.05).

### Intestine immunity-related gene expression analysis

**Figure 8:**
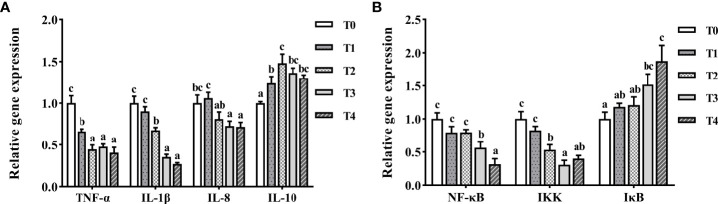
The expression profiles of inflammatory genes **(A)** and signaling pathway **(B)** in the intestine of *T. ovatus* fed diets with different dose taurine supplementation after 8 weeks. Mean values (n = 9) within values in the picture above with different superscripts are significantly different (*P* < 0.05).

The mRNA levels of *TNF-α*, *IL-1β*, and *IL-8* drastically decreased with increased taurine content (**Figure 8A**). The highest mRNA expression of *TNF-α* was observed in the control fish, which was much higher than those in the other experimental groups (*P* < 0.05). The highest mRNA expression of *IL-1β* was found in T0 and T1 fish, which was greatly higher than those of the other experimental groups (*P* < 0.05). With the increase of taurine content, *IL-10* mRNA expression first increased (0–0.8%) and then decreased (0.8–1.6%). The lowest mRNA expression of *IL-10* was found in the control fish, which was much lower than that in the other groups (*P* < 0.05). *NF-κb* and *IKK* mRNA expression decreased drastically with higher taurine content (**Figure 8B**). *NF-κB* mRNA expression with the exogenous addition of 1.2–1.6% taurine was considerably lower than that in the control group (*P* < 0.05). The *IKK* mRNA level was significantly higher in the T0 and T1 groups than in the other groups (*P* < 0.05). Conversely, *IκB* gene expression increased with increasing exogenous taurine supplementation in all test groups compared to that in the control group (*P* < 0.05).

### Streptococcus agalactiae challenge

**Figure 9:**
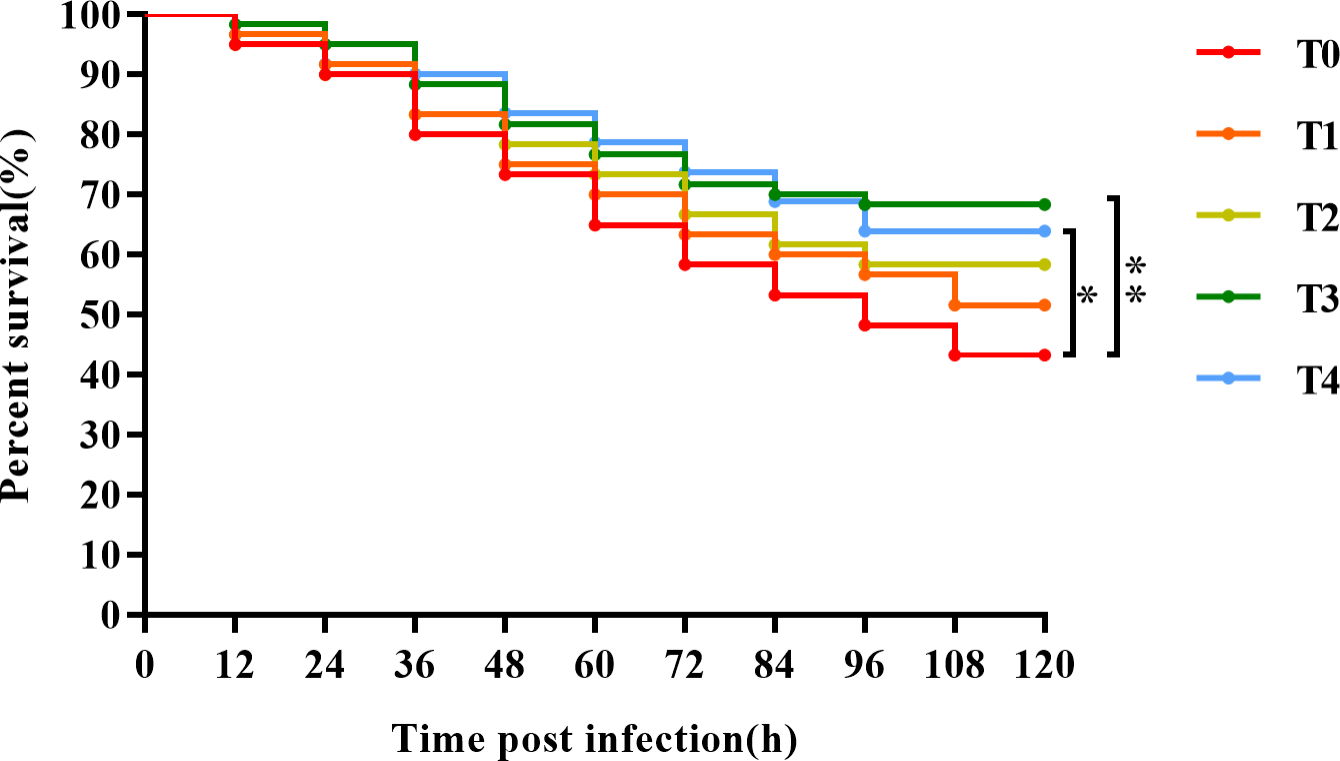
The Kaplan-Meier survival analysis of *T. ovatus* after *S. agalactiae* infection. Asterisks *, and ** indicate statistically significant difference between treated group and control group at *P* < 0.05, and *P* < 0.01, respectively.

As shown in **Figure 9**, after 120 h of *S. agalactiae* challenge, the survival rates of T0 (0), T1 (0.4%), T2 (0.8%), T3 (1.2%), and T4 (1.6%) fish were 48.33%, 56.67%, 63.33%, 66.67%, and 66.67%, respectively. Survival tended to increase with dietary taurine content. The survival rate of T3 fish was the highest and was significantly higher than that of the control group (*P* < 0.05), indicating that exogenous taurine can promote golden pompano immunity and pathogen resistance.

## Discussion

### Effects of exogenous taurine supplementation to low-fishmeal diets on the growth and body composition of juvenile golden pompano

Previous studies have shown that exogenous taurine supplementation to low-fishmeal diets may enhance the growth of fish (13–17). For example, exogenous taurine extremely increased the WGR and SGR of *Scophthalmus maximus L* (44), which greatly affected its growth. In the present study, exogenous taurine increased crude protein content and decreased HSI, improving WGR and growth performance in golden pompano, which is generally consistent with the findings of Ma et al. (37) from our laboratory. Various factors affect fish weight gain, including digestive enzyme activity, digestive tract characteristics, and growth-related hormone alterations.

Thyroxine is thought to promote growth and GH secretion (45). GH stimulates tissue growth, organismal anabolism, and protein synthesis by upregulating the synthesis and secretion of IGF-1 and IGF-2 (46). IGF-1 and IGF-2 play important roles in the growth and development of scleractinian fish (47). Studies on rats have shown that taurine can increase triiodothyronine and thyroxine levels by enhancing thyroid function (48). In addition, taurine can directly stimulate GH secretion in the brains of rats (49). In the present study, we observed that taurine supplementation increased the serum levels of growth-related parameters, such as GH, triiodothyronine, thyroxine, IGF-1, and IGF-2 in fish. NPY plays a vital physiological role in growth, development, reproduction, hormone release, and other activities of organisms. Studies on *Paralichthys olivaceus* have shown that NPY promotes GH release and can substantially increase feeding and growth rates (50). In rats, taurine was demonstrated to downregulate *NPY* expression within the hypothalamus, reducing appetite and food intake (51). In contrast, taurine intake greatly upregulated *GH* and *NPY* mRNA expression in the brain as well as *IGF-1* and *IGF-2* mRNA levels in the liver of the golden pompano. Thus, exogenous taurine intake not only enhances appetite in golden pompano but also promotes feeding and growth.

### Effects of exogenous taurine to low-fishmeal diets on oxidative stress resistance and non-specific immunity in juvenile golden pompano

Previous studies have demonstrated that taurine can increase antioxidant enzyme activity and reduce ROS levels in aquatic animals (18, 52). For example, exogenous taurine at 0.4–0.8% drastically increased the T-AOC, SOD, and GSH-PX activities while reducing MDA levels in *Eriocheir sinensis* (52). Similarly, the present study showed that exogenous taurine increased the T-AOC as well as CAT, GSH-PX, and SOD activities and intestinal gene expression, while decreasing MDA and ROS levels in golden pompano serum. Under normal conditions, ROS levels are mainly regulated by Nrf2 and its inhibitory partner Keap-1 (53), with increased transcription of *Nrf2* leading to a decrease in intracellular ROS (54). Nrf2 can also directly regulate *HO-1* promoter activity and rapidly increase antioxidant enzyme expression in fish. For example, taurine chloramine (TauCl), which is derived from taurine, can induce *HO-1* mRNA expression by activating the Nrf2/Keap-1/HO-1 axis, resulting in an enhanced antioxidant capacity (55, 56). Previous studies in our laboratory have shown that Nrf2/Keap-1/HO-1 is also active in the golden pompano and can provide protection against oxidative stress induced by acute ammonia exposure and copper (40, 41). In the present study, we observed that an increase in dietary taurine content significantly promoted and then suppressed intestinal *HO-1* and *Nrf2* mRNA levels, with significant upregulation of *Keap-1* mRNA levels, altogether providing evidence of an enhanced antioxidant capacity mediated *via* the Nrf2/Keap-1/HO-1 axis. However, it should be noted that SOD activity in serum and liver *HO-1* mRNA expression were not the highest and serum MDA levels were not at the lowest level under 1.6% (T4) taurine supplementation, thus suggesting that excessive exogenous taurine is not necessary.

Oxidative stress damages tissues and decreases the non-specific immune capacity (18). Serum LZM, C3, C4, IgM, IgA, and IgG are considered essential indicators of immune function (19, 57). Previous studies have indicated that taurine can increase IgM, C4, and C3 levels in juvenile *I. punctatus*, improve antioxidant capacity, and protect against oxidized fish oil (19). Exogenous taurine improved LZM activity, increased total immunoglobulin as well as C3 and C4 levels in *Carassius auratus*, reducing the adverse effects of high ammonia levels (57). Similarly, our study showed that exogenous taurine extremely increased serum LZM activity, increased C3, C4, IgM, IgA, and IgG levels, thus enhancing innate and adaptive immunity in the golden pompano.

### Effect of exogenous taurine supplementation to low-fishmeal diets on the intestine immunity of juvenile golden pompano

It is well known that the intestinal immune system, which consists of physical and immune barriers, is central to immune homeostasis in aquatic animals (21). Complete intestinal tissue structure is not only a prerequisite for digestion and absorption, but also a requirement for proper intestinal immune function (58). Taurine has been shown to effectively protect intestinal tissue integrity in fish. For example, a study on *Cyprinus carpi* showed that taurine increased villus length and goblet cell quantity (8). Under high-fat diet feeding, taurine addition increased *M. albus* (13) villus length and muscular thickness, resulting in a more regular arrangement of villi which was beneficial for maintaining intestinal health. Our study revealed that exogenous taurine increased intestinal villus length and muscular thickness, reduced the number of goblet cells, and protected the intestinal mucosal layer, thus maintaining intestinal barrier stability and promoting intestinal health in golden pompano.

When the proportion of fishmeal in feed is too low, oxidative stress is induced in the fish liver and intestine, leading to structural damage, reduced intestine immunity, and inflammation (8–11). As a defensive response against pathogen invasion and tissue damage (59), activation of the inflammatory response is mediated mainly *via* NF-κB, IκB, and IKK. In the classical NF-κB signaling pathway, IκB is degraded in response to IKK induction, resulting in NF-κB activation and the production of pro-inflammatory cytokines, such as IL-1β, IL-8, and TNF-α (60). In fish, the inflammatory response is regulated by a combination of anti-inflammatory cytokines (IL-10, TGF-β, etc.) and pro-inflammatory cytokines (IL-1β, IL-8, TNF-α, etc.) (61). As a functional amino acid, taurine can improve intestine immunity in fish and reduce aberrant inflammatory responses. For example, taurine can inhibit *NF-κB* expression in *I. punctatus*, downregulate pro-inflammatory cytokines (*IL-1β*, *IL-6*, *TNF-α*, and *IL-8*), and upregulate anti-inflammatory *TGF-β* mRNA expression, thereby improving intestine immunity and promoting intestine health (19). Similarly, our study showed that exogenous addition of 0.8–1.2% taurine (T2–T4) greatly reduced intestinal *IL-1β*, *IL-8*, and *TNF-α* mRNA levels, upregulating *IL-10*, which enhanced intestine immunity. This finding provides evidence that taurine protects intestinal health by enhancing anti-inflammatory activity.

This study also investigated the protective effects of taurine against *S. agalactiae*. Taurine exhibited a positive impact on the survival of golden pompano, with exogenous addition of 1.2–1.6% taurine extremely increasing survival after *S. agalactiae* challenge. Similarly, feeding *C. idella* with exogenous taurine effectively reduced the incidence of enteritis caused by *Aeromonas hydrophila* (23). Further, taurine can provide reasonable protection against mechanical stress in zebrafish (62) and *Vibrio alginolyticus* challenge (63). The protective effect of exogenous taurine bacterial challenge may stem from the enhanced intestinal antioxidant capacity of fish mediated *via* the Nrf2/Keap-1/HO-1 axis. Furthermore, exogenous taurine reduces the intestinal inflammatory response in fish by suppressing NF-κB signaling.

## Conclusions

In summary, moderate exogenous taurine improved upregulated growth-related gene expression, serum growth parameter levels, growth performance, and crude protein content in the golden pompano. The optimal taurine level in golden pompano diet was estimated at 1.18% *via* polynomial regression analysis using SGR. Moderate exogenous taurine also protected intestinal structural integrity, maintained intestinal physical barrier stability, activated Nrf2/Keap-1/HO-1 signaling pathway, increased intestinal antioxidant enzyme gene expression and serum antioxidant enzyme activity. Further, supplementation suppressed NF-κB signaling and intestinal pro-inflammatory cytokine gene expression, increased anti-inflammatory cytokine gene expression, and improved intestine immunity. Taurine protected juvenile golden pompanos after challenge with *S. agalactiae*. Our findings provide additional information and support for the rational use of taurine in healthy aquatic animal farming.

## Data availability statement

The data presented in the study are deposited in the Genbank repository, accession number OP292223.

## Ethics statement

The animal study was reviewed and approved by Animal Care and Use Committee of the South China Sea Fisheries Research Institute, Chinese Academy of Fishery Sciences(No. SCSFRI96-253).

## Author contributions

D-CZ designed the experiments, wrote the paper. J-XL conducted the experiment, wrote the paper. H-YG: Data curation. K-CZ: Visualization, Methodology. B-SL: Supervision, Methodology, Software. NZ: Visualization, Investigation. All authors contributed to the article and approved the submitted version.

## Funding

This research was financially supported by China Agriculture Research System of MOF and MARA (CARS-47), Central Public-Interest Scientific Institution Basal Research Fund of South China Sea Fisheries Research Institute CAFS (2021SD12), Central Public-interest Scientific Institution Basal Research Fund, CAFS (NO.2020TD29), Key Projects of Joint Fund for Regional Innovation and Development of NSFC (U20A2064), National Marine Genetic Resource Center, Guangdong Provincial Special Fund for Modern Agriculture Industry Technology Innovation Teams (2019KJ143), Financial Fund of Ministry of Agriculture and Rural Affairs of China (NHYYSWZZZYKZX2020).

## Conflict of interest

The authors declare that the research was conducted in the absence of any commercial or financial relationships that could be construed as a potential conflict of interest.

## Publisher’s note

All claims expressed in this article are solely those of the authors and do not necessarily represent those of their affiliated organizations, or those of the publisher, the editors and the reviewers. Any product that may be evaluated in this article, or claim that may be made by its manufacturer, is not guaranteed or endorsed by the publisher.
